# Characteristics of Autonomic Dysfunction in Parkinson’s Disease: A Large Chinese Multicenter Cohort Study

**DOI:** 10.3389/fnagi.2021.761044

**Published:** 2021-11-30

**Authors:** Zhou Zhou, Xiaoting Zhou, Xiaoxia Zhou, Yaqin Xiang, Liping Zhu, Lixia Qin, Yige Wang, Hongxu Pan, Yuwen Zhao, Qiying Sun, Qian Xu, Xinyin Wu, Xinxiang Yan, Jifeng Guo, Beisha Tang, Zhenhua Liu

**Affiliations:** ^1^Department of Neurology, Xiangya Hospital, Central South University, Changsha, China; ^2^Department of Geriatric Neurology, Xiangya Hospital, Central South University, Changsha, China; ^3^National Clinical Research Center for Geriatric Disorders, Xiangya Hospital, Central South University, Changsha, China; ^4^Department of Public Health, Xiangya School of Medicine, Central South University, Changsha, China; ^5^Center for Medical Genetics, School of Life Sciences, Central South University, Changsha, China; ^6^Key Laboratory of Hunan Province in Neurodegenerative Disorders, Central South University, Changsha, China

**Keywords:** Parkinson’s disease, non-motor symptoms, autonomic dysfunction, SCOPA-AUT, PD-MDCNC

## Abstract

Autonomic dysfunction (AutD) is one of the non-motor symptoms (NMSs) in Parkinson’s disease (PD). To investigate the prevalence and clinical features of AutD in Chinese patients with PD, a large multicenter cohort of 2,556 individuals with PD were consecutively involved in the Parkinson’s Disease and Movement Disorders Multicenter Database and Collaborative Network in China (PD-MDCNC) between January 1, 2017, and December 31, 2019. The assessment of AutD was performed using the Scale for Outcomes in Parkinson’s Disease for Autonomic Symptoms (SCOPA-AUT). The evaluation of motor symptoms and other NMSs were performed using well-established scales recommended by the Movement Disorder Society. We found that out of 2,556 patients with PD, 2,333 patients with PD (91.28%) had AutD. Compared with the group of patients with PD without AutD, the group of patients with PD with AutD had older age, older age of onset, longer disease duration, more severe motor symptoms, motor complications, and more frequent NMSs. As for partial correlation analysis, the total SCOPA-AUT score was significantly and positively associated with motor severity scales [Unified Parkinson’s Disease Rating Scale (UPDRS) total score] and some of the NMSs [Rapid Eye Movement Sleep Behavior Disorder Questionnaire (RBD), Epworth Sleepiness Scale, Hamilton Depression Scale], Fatigue Severity Scale, and Parkinson’s disease questionnaire. PD Sleep Scale was significantly and negatively correlated with AutD. With logistic regression analysis for potentially related factors, age, UPDRS total score, RBD, hyposmia, depression, and fatigue may be associated with PD with AutD. In conclusion, our multicenter cohort study reported the high prevalence of AutD in Chinese PD and revealed the associated factors of PD with AutD.

## Introduction

Parkinson’s disease (PD) is a complex neurodegenerative disorder with a multifactorial origin and varied clinical symptoms. Loss of neurons in the substantia nigra leads to a lack of dopamine in the striatum and the formation of intracellular inclusions containing α-synuclein aggregates that are neuropathological hallmarks of PD (Bloem et al., 2021). PD is characterized by motor symptoms, such as bradykinesia, resting tremor, rigidity, and postural instability, as well as non-motor symptoms (NMSs), including olfactory dysfunction, cognitive dysfunction, neuropsychiatric phenotypes, sleep disturbances, and autonomic dysfunction (AutD) (Schapira et al., 2017).

Dysfunction of the autonomic nervous system, AutD, can occur roughly 5–20 years earlier than the onset of typical PD motor symptoms (Pont-Sunyer et al., 2015; Postuma and Berg, 2016). Symptoms of AutD include, but are not limited to, constipation, urinary urgency, orthostatic hypotension, abnormal perspiration, and photophobia. AutD has been considered as a crucial factor that affects daily living and health-related quality of life (HRQoL) (Merola et al., 2017, 2018; Tomic et al., 2017). Furthermore, it may be associated with faster progression and shorter survival time in patients with PD (De Pablo-Fernandez et al., 2017). Despite the considerable understanding of PD symptoms, the exact pathogenesis of AutD is still largely unknown. Studies that demonstrate neuronal loss and abnormal aggregation of α-synuclein in the autonomic nervous system, including the dorsal motor nucleus of the vagus and sympathetic ganglia, may provide a pathological explanation for PD (Gelpi et al., 2014; Van Den Berge et al., 2021).

Given the high prevalence of AutD (14–84%) and its severe effect on the quality of life of patients, an increasing number of clinical studies have appeared in recent years (Wüllner et al., 2007; Arnao et al., 2015; Merola et al., 2017, 2018; Stankovic et al., 2019). Moreover, NMSs vary between Chinese and Western patients with PD based on the differences in sociodemographic factors, cultural backgrounds, and pharmacogenomics (Chen et al., 2012; Sauerbier et al., 2017). The Scale for Outcomes in PD for Autonomic Symptoms (SCOPA-AUT) has been used to assess the presence and frequency of autonomic symptoms in PD and has shown satisfactory reliability and validity (Visser et al., 2004). However, AutD is significantly diverse among populations (Qin et al., 2019; Wang et al., 2019). NMSs vary between Chinese and Western patients with PD due to disparities in sociodemographic factors, cultural backgrounds, and pharmacogenomics (Chen et al., 2012; Sauerbier et al., 2017). Therefore, the small-scale, monocentric cohorts previously reported in China may not comprehensively reflect the full scope of clinical features found in AutD (Qin et al., 2019; Wang et al., 2019). To overcome this limitation, our study aims to investigate the overall and individual prevalence of AutD, its clinical features, and the correlations among AutD and other PD symptoms in a large multicenter cohort.

## Materials and Methods

### Participants

**FIGURE 1:**
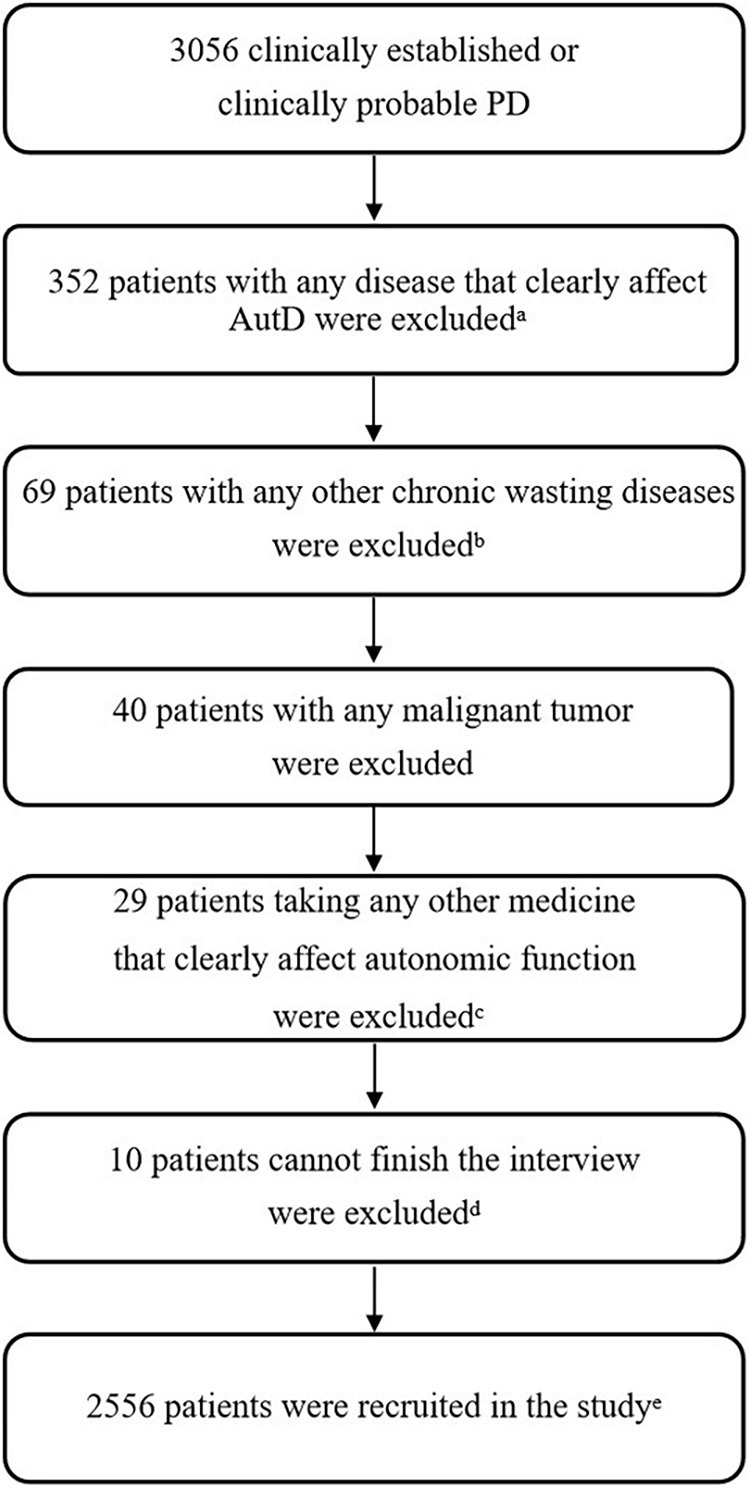
Flowchart of exclusion criteria. ^*a*^Other diseases that have severe autonomic complications, such as diabetes with ≥3 years, severe prostatic hypertrophy, and glaucoma. ^*b*^Chronic wasting diseases such as active tuberculosis, hyperthyroidism, chronic atrophic gastritis, renal failure, and anemia with hemoglobin ≤ 60 g/L. ^*c*^Experiencing autonomic side effects confirmed by doctors that were apparently associated with medication consumption that would affect the evaluation of AutD, such as severe diarrhea, constipation, fatigue, and sweating. ^*d*^10 patients cannot finish the interview due to cognitive impairment. ^*e*^If a patient met two or more of the above exclusion criteria, we took into count for only once. PD, Parkinson’s disease; AutD, autonomic dysfunction.

A total of 3,056 participants were involved from eight movement disorders centers in China from January 1, 2017, to December 31, 2019, as shown in Supplementary Table 1. Clinical diagnosis of PD was confirmed by at least one neurological specialist according to the MDS Clinical Diagnostic Criteria for PD (Postuma et al., 2015), including diagnoses of either clinically established PD or clinically probable PD. Exclusion criteria included (1) other diseases that have severe autonomic complications, such as diabetes with ≥3 years, severe prostatic hypertrophy, and glaucoma, (2) chronic wasting diseases such as active tuberculosis, hyperthyroidism, chronic atrophic gastritis, renal failure, and anemia with hemoglobin ≤ 60 g/L, (3) malignant tumors, and (4) experiencing autonomic side effects confirmed by doctors to be associated with medication consumption, such as severe diarrhea, constipation, fatigue, and sweating. Based on these criteria, 2,556 patients with PD were included in the study (Figure 1), and 352 patients with any disease that affects AutD, 69 patients with other chronic wasting diseases, 40 patients with cancer, 29 patients taking any other medicine that clearly affects AutD, and 10 patients who could not finish the interview were excluded.

This study was supported by the Parkinson’s Disease and Movement Disorders Multicenter Database and Collaborative Network in China (PD-MDCNC). The data were downloaded from the PD-MDCNC database on December 31, 2019. All patients signed informed consent before entering the study. The study was endorsed by the Ethics Committee of Central South University Xiangya Hospital.

### Clinical Assessment

Demographic and clinical characteristics were collected as described in our previous study, including age, gender, body mass index (BMI), age of onset, disease duration, history of smoking, alcohol intake, drinking tea, and pesticide exposure (Zhao et al., 2020). Six autonomic function-related domains were recorded from SCOPA-AUT, including gastrointestinal, urinary, cardiovascular, thermoregulatory, pupillomotor, and sexual domains. Clinical examinations of motor symptoms were performed based on the Unified Parkinson’s Disease Rating Scale (UPDRS) and Hoehn and Yahr (H&Y) scale, defining motor subtypes as either tremor dominant (TD), postural instability/gait difficulty (PIGD), or indeterminate group (Stebbins et al., 2013). Motor complications such as dyskinesia, freezing of gait (FOG), and wearing-off were diagnosed by clinicians, and the severity of each was evaluated using UPDRS-IV part A, New Freezing of Gait Questionnaire (NFOG-Q), and the 9-Item End-of-Dose Wearing-Off Questionnaire (WOQ-9), respectively. Levodopa equivalent daily dose (LEDD) was calculated based on a commonly used method (Tomlinson et al., 2010).

In addition to motor symptoms, we evaluated a broad range of NMSs based on the Non-Motor Symptoms Scale (NMSS), Mini-Mental State Examination (MMSE), Rapid Eye Movement Sleep Behavior Disorder Questionnaire-Hong Kong (RBDQ-HK), Epworth Sleepiness Scale (ESS), sleeping performance by Parkinson’s Disease Sleep Scale (PDSS), Hyposmia Rating Scale (HRS), Hamilton Depression Scale (HAMD-17), Fatigue Severity Scale (FSS), and Cambridge-Hopkins questionnaire for restless legs syndrome (CH-RLSq). The quality of life was assessed using Parkinson’s Disease Questionnaire-39 item version (PDQ-39). Cognitive impairment was defined as an MMSE total score equal to or less than 24 (Katzman et al., 1988). Hyposmia was defined as a total HRS scale less than 22.5 (Millar Vernetti et al., 2012). Rapid eye movement sleep behavior disorder (RBD) was defined as either a total RBDQ-HK scale not less than 18 or a RBDQ-HK part II score not less than 13 (Li et al., 2010). Restless legs syndrome (RLS) was defined based on the diagnostic criteria of CH-RLSq (Allen et al., 2009; Walters et al., 2014). Excessive daytime sleepiness (EDS) was defined as a total ESS score higher than 9 (Johns, 1991). Fatigue was defined as a FSS mean score not less than 3.3 (Brown et al., 2005). Depression was defined as a total HAMD-17 score higher than 7 (Montgomery and Asberg, 1979).

The evaluations in this study were only conducted once. Both a clinician and a researcher guided the assessments and recorded the results of basic information collection and scale evaluations. Along with patients, and at least one of their primary family caregivers participated in the assessment as well. Before carrying out the assessment, all researchers were trained to ensure equal understanding of the scales used as well as the methods and phrasing for clinical data collection.

### Definition of Autonomic Dysfunction

Autonomic dysfunction was defined as a score greater than or equal to one in SCOPA-AUT. A score of zero was regarded as normal autonomic function. Each of the six autonomic domains was categorized as impaired when at least one of the related items was rated. Referring to Merola, patients were further divided into three subgroups according to the number of affected domains: without AutD, with single-domain AutD, and with multiple-domain AutD (Merola et al., 2018).

### Statistical Analysis

Continuous variables were analyzed based on one-way ANOVA or using nonparametric tests. Categorical variables were analyzed using the chi-square test.

Partial correlation analysis was used to analyze relationships between SCOPA-AUT score and other clinical variables while better controlling other potential random variables. This analysis included the SCOPA-AUT total score and its 6 subdomains adjusted by age, gender, and disease duration, as well as 12 variables including body BMI, age of onset, LEDD, UPDRS total score, MMSE, RBDQ-HK, ESS, PDSS, HRS, HAMD, FSS, and PDQ-39.

For binary logistic regression analysis, as many variables were converted into categorical variables as possible. A total of 21 variables, including age, gender, BMI, and age of onset, were divided into two groups (earlier or later than 50 years old), disease duration, LEDD, UPDRS total score, motor subtypes, H&Y stage (H&Y was divided into two groups, i.e., stage 1–2.5 and stage 3–5), with or without dyskinesia, wearing-off, FOG, with or without cognition impairment, RBD, EDS, RLS, hyposmia, depression, fatigue, PDSS score, and PDQ-39 were introduced into the model to diagnose the multicollinearity before running the logistic regression analysis. The variance inflation factor (VIF) in the SPSS software was used as a predictor for multicollinearity. Variable with a VIF score less than 10.0 is considered as mild multicollinearity. Among the 21 variables tested, the wearing-off was removed because of its VIF score higher than 10. Statistical significance was defined as *p* < 0.05. All data were analyzed with the IBM SPSS Statistics version 23.0 (IBM Corp., United States) software.

## Results

### Overview

In the cohort of 2,556 participants with PD (52.23% men and 47.77% women), the mean age was 61.03 ± 10.15 years. The mean age of PD onset was 55.67 ± 10.79 years, with an average disease duration of 5.39 ± 4.73 years. The mean LEDD was 314.47 ± 264.12 mg. Detailed information is shown in Table 1.

**TABLE 1:** Demographic and clinical characteristics of patients with PD.

**Characteristics**	**Total (*n* = 2,556)**	**Median (25–75% interquartile)**
Age	61.03 ± 10.15	62.00 (54.00–68.00)
Gender ratio (male/total)	52.23%	–
BMI	23.51 ± 4.62	23.51 (20.13–26.67)
Smoking history	25.01%	–
Alcohol intake history	25.14%	–
Drinking tea history	13.44%	–
Pesticide exposure history	11.13%	–
Age of onset	55.67 ± 10.79	56.00 (48.00–63.00)
Disease duration	5.39 ± 4.73	4.00 (2.00–7.00)
LEDD	314.47 ± 264.12	300.00 (150.00–437.50)
Drug-naïve patients	17.04%	
Levodopa	77.28%	–
Non-ergot DR agonists	52.94%	–
COMT inhibitors	7.14%	–
MAO-B inhibitors	10.47%	–
Anticholinergic drugs	15.25%	–
Amantadine	11.36%	–
UPDRS I	2.39 ± 2.06	2.00 (1.00–4.00)
UPDRS II	11.88 ± 6.56	10.00 (8.00–15.00)
UPDRS III	27.20 ± 15.35	25.00 (16.00–35.00)
UPDRS total score	42.96 ± 22.57	39.00 (27.00–55.00)
Tremor score	5.26 ± 4.50	4.00 (2.00–7.00)
Postural and gait score	4.43 ± 3.27	4.00 (2.00–6.00)
Motor subtypes	39.49/13.74/46.77	–
TD/Indeterminate/PIGD (%)		
H&Y stage	2.0 (1.5–3)	–
1	15.38%	–
1.5	13.54%	–
2	22.30%	–
2.5	21.13%	–
3	21.95%	–
4	4.54%	–
5	1.17 %	–
Dyskinesia	16.55%	–
Wearing-off	23.06%	–
FOG	24.78%	–
NMSS	36.06 ± 27.20	31.00 (16.00–50.00)
MMSE	26.37 ± 3.69	27.00 (25.00–29.00)
RBDQ-HK	16.89 ± 17.11	11.00 (3.00–27.00)
ESS	7.80 ± 6.42	7.00 (2.00–12.00)
PDSS	115.22 ± 27.51	121.00 (102.00–135.00)
HRS	19.62 ± 6.29	24.00 (18.00–24.00)
HAMD	6.08 ± 6.04	5.00 (1.00–9.00)
RLS	10.61%	–
FSS	45.87 ± 19.09	48.00 (32.00–64.00)
PDQ-39	29.98 ± 26.44	23.00 (9.00–43.00)

*Quantitative data were expressed as mean ± SD. Categorical variables were expressed as percentages. PD, Parkinson’s disease; BMI, body mass index; LEDD, Levodopa equivalent daily dose; DR, dopamine receptor; COMT, catechol-*O*-methyltransferase; MAO-B, monoamine oxidase-B; UPDRS, Unified Parkinson’s Disease Rating Scale; TD, tremor dominant; PIGD, postural instability, and gait difficulty; H&Y, Hoehn and Yahr; FOG, freezing of gait; NMSS, Non-Motor Symptoms Scale; MMSE, Mini-Mental State Examination; RBDQ-HK, Rapid Eye Movement Sleep Behavior Disorder Questionnaire-Hong Kong; ESS, Epworth Sleepiness Scale; PDSS, Parkinson’s Disease Sleep Scale; HRS, Hyposmia Rating Scale; HAMD, Hamilton Depression Scale; RLS, restless legs syndrome; FSS, Fatigue Severity Scale; PDQ-39, Parkinson’s Disease Questionnaire-39 item version.*

A large majority of patients in this cohort experienced autonomic dysfunction (2,333 out of 2,556 or 91.28%). The average score of SCOPA-AUT was 9.21 ± 7.39, with a median (25–75% interquartile) of 8 (3–13). The most frequently affected domain in PD with AutD was gastrointestinal (72.30%), followed by urinary (71.05%), thermoregulatory (44.25%), cardiovascular (23.87%), sexual (21.79%), and pupillomotor dysfunction (15.61%). The top five most frequently involved symptoms were nocturia (63.69%), straining for defecation (49.41%), constipation (45.11%), sialorrhea (44.95%), and urinary urgency (37.21%). The top five least frequently involved symptoms were fecal incontinence (2.27%), syncope (2.54%), dysphagia (10.33%), early abdominal fullness (11.35%), and hyperhidrosis during the night (15.14%). An overview of each SCOPA-AUT item is summarized in Supplementary Table 2.

### Clinical Characteristics of Autonomic Dysfunction

As shown in Table 2, we compared the demographic and clinical characteristics of patients with PD who fall under the without AutD group, single-domain AutD group, or multiple-domain AutD group. Statistically significant differences (*p* < 0.05) were observed across age, BMI, history of pesticide exposure, age of onset, disease duration, LEDD, UPDRS I, II, III score, UPDRS total score, postural and gait score, the motor subtypes, H&Y stage, dyskinesia, wearing-off, FOG, MMSE, RBDQ-HK, ESS, PDSS, HRS, HAMD, RLS, FSS, and PDQ-39 score.

**TABLE 2:** Demographic and clinical characteristics of patients with PD without and with single-domain and multiple-domain AutD.

**Characteristics**	**Without AutD (*n* = 223)**	**Single-Domain AutD (*n* = 387)**	**Multiple-Domain AutD (*n* = 1946)**	**Total *p-value***	**Multiple comparisons adjusted *p-value*^∗^**		
					***p-_*WOA*_ _*VS SDA*_***	***p-_*WOA*_ _*VS MDA*_***	***p-*_*SDA*_ _*VS MDA*_**
Age	55.02 ± 9.82	58.70 ± 10.13	62.18 ± 9.87	**<0.001**	**<0.001**	**<0.001**	**<0.001**
Gender ratio (male)	47.53%	51.94%	52.83%	0.323	–	–	–
BMI	21.16 ± 4.51	22.53 ± 4.51	23.96 ± 4.54	**<0.001**	**0.002**	**<0.001**	**<0.001**
Smoking history	21.15%	25.00%	25.44%	0.400	–	–	–
Alcohol intake history	20.28%	23.95%	25.91%	0.170	–	–	–
Drinking tea history	9.13%	13.87%	13.86%	0.147	–	–	–
Pesticide exposure history	4.48%	10.44%	12.05%	**0.003**	**<0.05**	**<0.05**	>0.05
Age of onset	51.26 ± 11.24	54.51 ± 10.71	56.41 ± 10.62	**<0.001**	**0.004**	**<0.001**	**0.003**
Disease duration	3.96 ± 4.47	4.21 ± 3.69	5.79 ± 4.88	**<0.001**	0.202	**<0.001**	**<0.001**
LEDD	250.02 ± 225.56	281.01 ± 241.23	328.79 ± 271.02	**<0.001**	0.310	**<0.001**	**0.005**
UPDRS I	1.63 ± 1.54	1.83 ± 1.71	2.59 ± 2.13	**<0.001**	0.715	**<0.001**	**<0.001**
UPDRS II	8.25 ± 5.09	9.43 ± 5.03	12.78 ± 6.71	**<0.001**	**0.012**	**<0.001**	**<0.001**
UPDRS III	20.62 ± 12.77	22.97 ± 13.47	28.79 ± 15.61	**<0.001**	0.107	**<0.001**	**<0.001**
UPDRS total score	31.22 ± 18.07	35.21 ± 18.66	45.84 ± 22.96	**<0.001**	**0.033**	**<0.001**	**<0.001**
Tremor score	4.54 ± 3.74	4.92 ± 4.01	5.41 ± 4.66	0.076	–	–	–
Postural and gait score	3.04 ± 2.41	3.36 ± 2.66	4.80 ± 3.37	**<0.001**	0.734	**<0.001**	**<0.001**
Motor subtypes	46.19/16.59/37.22	46.77/14.99/38.24	37.31/13.16/49.54	**<0.001**	–	–	–
TD/Indeterminate/PIGD (%)							
H&Y stage^*^	1.5 (1–2.5)	2 (1–2.5)	2.5 (2–3)	**<0.001**	0.119	**<0.001**	**<0.001**
<3	86.10%	83.72%	68.50%	–	–	–	–
≥3	13.90%	16.28%	31.50%	–	–	–	–
Dyskinesia	10.81%	10.85%	18.34%	**<0.001**	>0.05	**<0.05**	**<0.05**
Wearing-off	14.22%	16.09%	25.41%	**<0.001**	>0.05	**<0.05**	**<0.05**
FOG	13.57%	17.73%	27.33%	**<0.001**	>0.05	**<0.05**	**<0.05**
NMSS	16.04 ± 19.51	21.70 ± 18.04	41.22 ± 27.39	–	–	–	–
MMSE	27.66 ± 3.39	27.16 ± 3.27	26.07 ± 3.76	**<0.001**	0.085	**<0.001**	**<0.001**
RBDQ-HK	8.25 ± 11.85	12.15 ± 14.79	18.82 ± 17.56	**<0.001**	**0.003**	**<0.001**	**<0.001**
ESS	5.78 ± 5.90	6.51 ± 6.13	8.29 ± 6.45	**<0.001**	0.434	**<0.001**	**<0.001**
PDSS	124.12 ± 28.45	124.57 ± 24.78	112.32 ± 27.29	**<0.001**	1.000	**<0.001**	**<0.001**
HRS	21.53 ± 4.84	20.58 ± 5.74	19.21 ± 6.48	**<0.001**	0.452	**<0.001**	**<0.001**
HAMD	3.24 ± 5.00	4.15 ± 5.19	6.79 ± 6.12	**<0.001**	**0.038**	**<0.001**	**<0.001**
RLS	8.42%	6.70%	11.63%	**0.013**	>0.05	>0.05	**<0.05**
FSS	37.41 ± 16.97	38.24 ± 18.34	48.36 ± 18.79	**<0.001**	1.000	**<0.001**	**<0.001**
PDQ-39	17.57 ± 20.30	19.86 ± 21.74	33.35 ± 26.97	**<0.001**	0.470	**<0.001**	**<0.001**

Quantitative data were expressed as means ± SD. Categorical variables were expressed as percentages. Bold value means *p* < 0.05. *H&Y stage was expressed as median (25–75% interquartile), subordinate levels were expressed as percentages. AutD, autonomic dysfunction; WOA, without AutD; SDA, single-domain AutD; MDA, multiple-domain AutD.

Next, we performed multiple comparisons to further elucidate the differences among groups. The results showed that age, BMI, age of onset, disease duration, scores of UPDRS II, total UPDRS, RBDQ-HK, and HAMD tended to increase progressively along with the increasing number of affected SCOPA-AUT domains. The above factors showed statistical significance between each multiple comparison of the subgroups (*p* < 0.05). In addition, comparison of disease duration, LEDD, UPDRS I, III score, postural and gait score, the motor subtypes, H&Y stage, the incidence of wearing-off, MMSE, ESS, PDSS, HRS, FSS, and PDQ-39 scores showed statistical significance between without AutD group and the multiple-domain AutD group. However, we did not detect any statistical significance between the without AutD group and the single-domain AutD group. An overview of the comparisons among the three subgroups is summarized in Table 2.

The NMSS covers a broad range of non-motor symptoms, encompassing 9 domains and 30 items (Chaudhuri et al., 2007). Three of those domains, including gastrointestinal, urinary, and sexual function domains, are related to autonomic function. To evaluate the autonomic dysfunctions using another alternative scale, as well as to compare its authenticity and reliability with the SCOPA-AUT scale, we performed comparison analysis with different domains of NMSS among AutD subgroups as shown in Supplementary Table 3 and Supplementary Figure 1. The average score of every NMSS domain increases as the affected AutD domains increase. In addition, the scores of the three autonomic-related domains in NMSS were significantly correlated with that in SCOPA-AUT, which indicates that the scoring system for assessing autonomic symptoms was consistent with both SCOPA-AUT and NMSS scales.

To control for the heterogeneity of patient timelines, we stratified PD cases into three subgroups based on disease duration (less than 3, 3–8, and more than 8 years) and four subgroups based on the age of onset, as shown in Supplementary Tables 4, 5, respectively. The overall SCOPA-AUT score as well as the scores in gastrointestinal, urinary, thermoregulatory, and male sexual domains increased as the disease progressed. However, the cardiovascular and pupillomotor functions were less likely to be associated with disease progression. Additionally, we found that gastrointestinal, urinary, cardiovascular dysfunctions were prone to occur in patients with PD with older disease onset. Conversely, male sexual dysfunctions were more prominent in early-onset patients with PD. The thermoregulatory and pupillomotor functions did not show any significant difference among different diseases of onset subgroups.

### Correlation Between Scale for Outcomes in Parkinson’s Disease for Autonomic Symptoms and Clinical Factors

**FIGURE 2:**
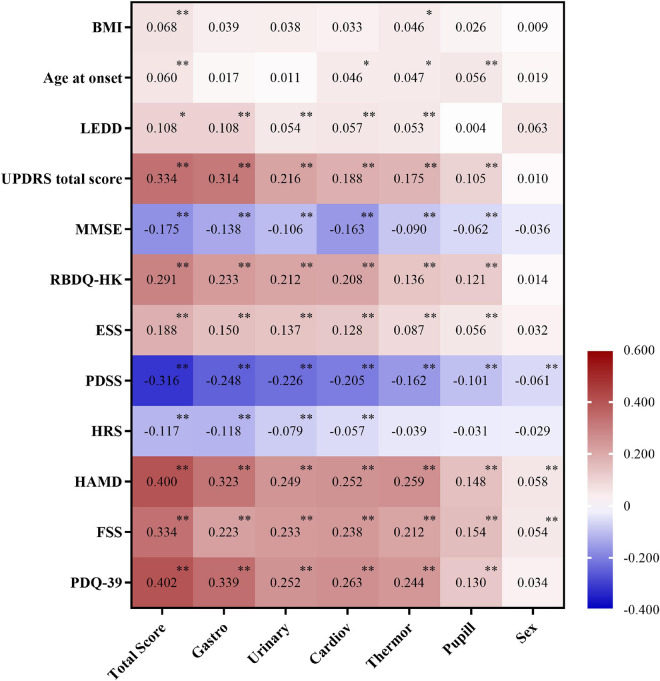
Correlation between Scale for Outcomes in Parkinson’s disease for autonomic symptoms (SCOPA-AUT) and clinical factors adjusted by age, gender, and disease duration. Number indicates partial correlation coefficient, 0.01 ≤ ^∗^*p* < 0.05, and ^∗∗^*p* < 0.01. BMI, body mass index; LEDD, Levodopa equivalent daily dose; UPDRS, Unified Parkinson’s Disease Rating Scale; H&Y, Hoehn and Yahr; MMSE, Mini-Mental State Examination; RBDQ-HK, Rapid Eye Movement Sleep Behavior Disorder Questionnaire-Hong Kong; ESS, Epworth Sleepiness Scale; PDSS, Parkinson’s Disease Sleep Scale; HRS, Hyposmia Rating Scale; HAMD, Hamilton Depression Scale; FSS, Fatigue Severity Scale; PDQ-39, Parkinson’s Disease Questionnaire-39 item version; Gastro, gastrointestinal dysfunction; Urinary, urinary dysfunction; Cardiov, cardiovascular dysfunction; Thermor, thermoregulatory dysfunction; Pupill, pupillomotor dysfunction; Sex, sexual dysfunction.

The presence and severity of AutD can be affected by diverse clinical factors as some autonomic phenotypes may occur simultaneously with other clinical symptoms. To thoroughly investigate the correlation between SCOPA-AUT and other PD clinical manifestations, we employed partial correlation analysis investigating the SCOPA-AUT total score and its six subdomains (gastrointestinal, urinary, cardiovascular, thermoregulatory, pupillomotor, and sexual domains). The total SCOPA-AUT score was significantly and positively associated with the motor severity scales (UPDRS total score) and some of the non-motor symptoms, such as ESS and RBD. Meanwhile, the overall score of AutD and almost all the subdomains of AutD, except for the sexual domains, showed a significant correlation with the HAMD, fatigue scale, and PDQ-39, all of which were commonly used methods for the assessment of the quality of life. The gastrointestinal domain turned out to be the most significant contributor among all the domains of the SCOPA-AUT scale. The PDSS, MMSE, and HRS scores, in which lower scores indicate more severe symptoms, were also significantly and negatively correlated with the AutD total score and most of the subdomains. Details are shown in Figure 2. Together these results emphasize that autonomic dysfunctions are a key factor affecting the quality of life of patients with PD.

### Factors Associated With Autonomic Dysfunction

Since the occurrence of AutD is associated with multiple clinical symptoms as shown in Figure 2, a multivariate logistic regression analysis was performed to investigate potential predictors for AutD. NMSS was removed from the logistic regression model as it has shared domains with autonomic dysfunction and was considered to be a confounder of AutD. Additionally, wearing-off was deleted from the variable list due to its high risk of multicollinearity. Detailed results are shown in Supplementary Table 6. Variables that are not present in the logistic regression model are shown in Supplementary Table 7. The logistic regression model demonstrated that age (OR = 1.055, 95% CI: 1.036–1.074), UPDRS total score (OR = 1.022, 95% CI: 1.011–1.034), RBD (OR = 2.138, 95% CI: 1.331–3.434), hyposmia (OR = 1.624, 95% CI: 1.090–2.420), depression (OR = 1.789, 95% CI: 1.049–3.052), and fatigue (OR = 1.682, 95% CI: 1.086–2.604) may be linked to PD with AutD. All related factors are shown in Table 3. In conjunction with the correlation analysis shown in Figure 2, the consistent positive correlations between AutD and either RBD, depression, or fatigue indicate that these symptoms are inclined to occur simultaneously, which may suggest a similar underlying mechanism between AutD and these symptoms. Differing from the partial correlation analysis, age of onset, motor symptom severity, and motor complications were not significant AutD predictors in the multivariate logistic regression model.

**TABLE 3:** Multivariate logistic regression analysis showing factors that related to PD with AutD.

**Variables**	**Odds ratio**	**95% CI**	***p-value***
Age	1.055	1.036–1.074	<0.001
UPDRS total score	1.022	1.011–1.034	<0.001
RBD (with vs without)	2.138	1.331–3.434	0.002
Hyposmia (with vs without)	1.624	1.090–2.420	0.017
Depression (with vs without)	1.789	1.049–3.052	0.033
Fatigue (with vs without)	1.682	1.086–2.604	0.020

*RBD, rapid eye movement sleep behavior disorder.*

## Discussion

Autonomic dysfunction can present as a wide variety of symptoms, varying greatly between individuals, and is often not well recognized during routine clinical visits, which may be one of the reasons for its diverse prevalence and characteristics from different ethnicities and cohorts (Shulman et al., 2002). The most frequent AutD were constipation (72%), followed by nocturia (65%) as it is summarized in a review manuscript (Chen et al., 2012). The PRIAMO study, which aimed to study the prevalence and presentation of NMSs amongst 1,000 patients with PD, found that more than 98% of the patients had at least one NMS, illustrating the importance of studying these phenotypes in patients with PD (Barone et al., 2009). In a prospective study that involved 101 PD cases, the prevalence of sleep disturbance was 43% as evaluated using the Pittsburgh Sleep Quality Inventory scale. However, neurologists failed to recognize sleep disturbance in 40% of patients in routine clinical examinations (Shulman et al., 2002). In this study, we performed a large-scale, multicenter analysis of AutD in Chinese patients with PD, who seem to be different from the results shown in Western studies. Significant differences in age, gender, motor symptoms, motor complications, and other NMSs domains were shown between patients with PD with and without AutD.

In this study, we found that the most prominently affected autonomic symptoms were urinary dysfunctions with a mean score of 3.40, followed by gastrointestinal dysfunctions. Similar results were observed in a recent study. Qin et al. (2019) in another Chinese cohort including 108 patients with PD at the early and middle stages reported that the most common gastrointestinal AutD were dry mouth (59.26%), followed by constipation (49.07%) and dysgeusia (37.04%). The most common autonomic dysfunctions reported in the PRIAMO study among the non-motor symptoms were fatigue and insomnia (37%) and urgency and nocturia (35%) (Barone et al., 2009). The disparity between the prevalence of AutD in China and western countries may be related to sample size, disease duration, and stage, in addition to racial diversity.

From the partial correlation analysis, both the overall and subdomains of AutD were significantly correlated with motor symptom parameters, such as LEDD and UPDRS total score. Studies have shown similar results that patients with PD with AutD are usually accompanied by severe motor symptoms (Verbaan et al., 2007; Arnao et al., 2015). Besides, AutD was also correlated with most of the non-motor symptoms in the correlation analysis.

From the logistic regression analysis, our results suggested that older age and higher UPDRS total score, consistent with the partial correlation analysis, was related to AutD (Verbaan et al., 2007; Wüllner et al., 2007). Among all the non-motor symptoms, RBD, hyposmia, depression, and fatigue turned out to be potential strong predictors for AutD. RBD, hyposmia, and depression were associated with AutD (Braak et al., 2003; Boeve et al., 2007). Another British cohort including 1,746 early patients with PD (within 3.5 years from PD diagnoses) found that RBD, depression, EDS, and PIGD subtype were significantly correlated with autonomic severity evaluated using SCOPA-AUT scale (Malek et al., 2017). Their study partially validated the finding in our cohort. The similar results between their study and our Chinese cohort showed that AutD was strongly correlated with these early PD non-motor symptoms. This provided a possible linkage between potential PD pathological change patterns and autonomic disorders progression, but it still needs verification from other clinical cohorts in the future. The hypothesis that NMS may all have mutual influence and permeation can partially explain our result. Several studies have demonstrated that patients with RBD may be affected by an underlying synucleinopathy pathological process that links to AutD, especially orthostatic hypotension and gastrointestinal dysfunction (Ferini-Strambi et al., 2014; Lee et al., 2015; Knudsen et al., 2019). Therefore, RBD and AutD may interact with each other, yielding individual and combined negative effects on PD (Li et al., 2017; Pilotto et al., 2019). Taken as a whole, the obvious significance of the number of and severity of NMS was shown in the PD with AutD group. Our study discovered that the more severe AutD, the worse the PDQ-39 score was, indicating that severe autonomic nervous symptoms may have a great impact on the quality of life of patients.

The scientific nature of the research is a large, multicenter cohort from eight movement disorders centers, and the integrity and coverage of the data collected were satisfactory. Thus, the study relatively reflected authentic clinical characteristics of Chinese PD with AutD. However, there are limitations to our study. First, as a cross-sectional study, it only explains the relationship rather than causality between related factors and AutD. A large longitudinal cohort is warranted to thoroughly elucidate the causal relationship in the future. Second, most of the data in this study are collected by a series of clinical scales to reflect the features of PD with AutD. Although the scales included in the study were recommended by MDS, the relatively subjective way of scoring and rating for individuals is biased. More objective examinations or intelligence-based evaluation in large cohorts may be the future direction to minimize bias.

Of note, the average score of SCOPA-AUT in our study was lower than other Asian studies (Matsushima et al., 2014; Kim et al., 2017; Wang et al., 2019). This may be due to the younger age of patients and stricter inclusion criteria in our cohorts, which excluded patients with PD diagnosed with diseases that affect AutD. Furthermore, our study showed a lower prevalence of symptoms in the sexual domain compared with other Asian studies, which may be due to the hesitation of Chinese patients to answer questions about sex due to cultural norms.

In summary, we studied the prevalence and clinical characteristics of AutD in a large cohort of Chinese patients with PD, which provided an overview of the yet poorly understood Chinese PD with AutD. In the future, long-term follow-up studies will be needed to verify the patterns of AutD in Chinese patients with PD.

## Data Availability Statement

The raw data supporting the conclusions of this article will be made available by the authors, without undue reservation.

## Ethics Statement

The studies involving human participants were reviewed and approved by Ethics Committee of Xiangya Hospital of Central South University. The patients/participants provided their written informed consent to participate in this study.

## Author Contributions

ZZ, XTZ, BT, and ZL: research project–conception and organization and statistical analysis–design and execution of the study. ZZ, XTZ, XXZ, YX, LQ, LZ, YW, HP, YZ, QS, QX, XY, and JG: research project execution and experimental works. ZZ and XTZ: writing the first draft of the manuscript. XW, BT, and ZL: statistical analysis and manuscript–review and critique. All authors read and approved the final manuscript.

## Conflict of Interest

The authors declare that the research was conducted in the absence of any commercial or financial relationships that could be construed as a potential conflict of interest.

## Publisher’s Note

All claims expressed in this article are solely those of the authors and do not necessarily represent those of their affiliated organizations, or those of the publisher, the editors and the reviewers. Any product that may be evaluated in this article, or claim that may be made by its manufacturer, is not guaranteed or endorsed by the publisher.

## Supplementary Material

Supplementary Figure 1 Subdomain correlation of NMSS with SCOPA-AUT. Number indicates partial correlation coefficient, 0.01 ≤ ^∗^*p* < 0.05, and ^∗∗^*p* < 0.01. NMSS, Non-Motor Symptoms Scale.
