# Supplementary figures and images for: Characteristics of Autonomic Dysfunction in Parkinson’s Disease: A Large Chinese Multicenter Cohort Study
Source: Front Aging Neurosci. 2021 Nov 30;13:761044. doi: 10.3389/fnagi.2021.761044 (PMC8670376; doi:10.3389/fnagi.2021.761044)

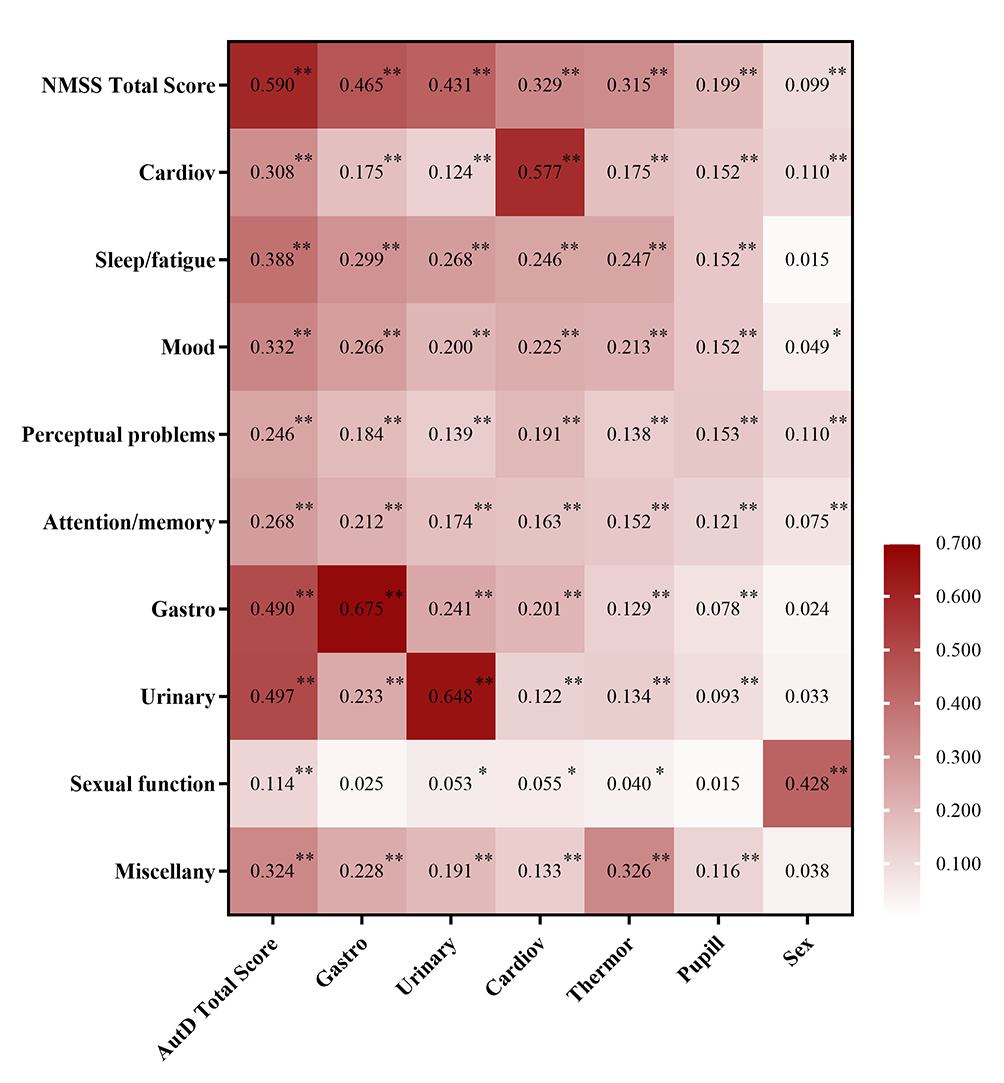

Supplement: Supplementary Figure 1 — Subdomain correlation of NMSS with SCOPA-AUT. Number indicates partial correlation coefficient, 0.01 ≤ ∗p < 0.05, and ∗∗p < 0.01. NMSS, Non-Motor Symptoms Scale. [file Image_1.TIF]
