# Supplementary material for: Characteristics of Autonomic Dysfunction in Parkinson’s Disease: A Large Chinese Multicenter Cohort Study
Source: Front Aging Neurosci. 2021 Nov 30;13:761044. doi: 10.3389/fnagi.2021.761044 (PMC8670376; doi:10.3389/fnagi.2021.761044)
Supplement: Supplementary file 2 [file Table_1.DOCX]

**Supplementary Table 1：Detail information for the eight centers in our recruitment**

| **Center** | **City** | **Province** |
| --- | --- | --- |
| Xiangya Hospital, Central South University | Changsha | Hunan |
| The Second Xiangya Hospital, Central South University | Changsha | Hunan |
| The Third Xiangya Hospital, Central South University | Changsha | Hunan |
| The First Affiliated Hospital of University of South China | Hengyang | Hunan |
| Hainan General Hospital | Haikou | Hainan |
| Wuhan Union Hospital | Wuhan | Hubei |
| The First Affiliated Hospital of Zhengzhou University | Zhengzhou | Henan |
| Affiliated Hospital of Zunyi Medical University | Zunyi | Guizhou |
