# Supplementary material for: Characteristics of Autonomic Dysfunction in Parkinson’s Disease: A Large Chinese Multicenter Cohort Study
Source: Front Aging Neurosci. 2021 Nov 30;13:761044. doi: 10.3389/fnagi.2021.761044 (PMC8670376; doi:10.3389/fnagi.2021.761044)
Supplement: Supplementary file 3 [file Table_2.DOCX]

**Supplementary Table 2：Overview of AutD symptom in SCOPA-AUT**

| **Items** | **Mean±SD** | **Median*** | **Items** | **Mean±SD** | **Median*** |
| --- | --- | --- | --- | --- | --- |
| **Gastrointestinal dysfunction** | 3.25±3.22 | 2 (0～6) | **Cardiovascular dysfunction** | 0.53±1.14 | 0 (0～0) |
| Difficulty swallowing/choked | 0.29±0.57 | 0 (0～0) | Light-headed when standing up | 0.28±0.62 | 0 (0～0) |
| Sialorrhea | 0.66±0.86 | 0 (0～1) | Light-headed when standing for some time | 0.21±0.55 | 0 (0～0) |
| Dysphagia | 0.13±0.43 | 0 (0～0) | Syncope | 0.03±0.20 | 0 (0～0) |
| Early abdominal fullness | 0.16±0.50 | 0 (0～0) | **Thermoregulatory dysfunction** | 1.35±2.02 | 0 (0～2) |
| Constipation | 0.96±1.18 | 0 (0～2) | Hyperhidrosis during the day | 0.42±0.80 | 0 (0～0) |
| Straining for defecation | 1.01±1.17 | 0 (0～2) | Hyperhidrosis during the night | 0.25±0.64 | 0 (0～0) |
| Fecal incontinence | 0.03±0.23 | 0 (0～0) | Cold intolerance | 0.33±0.71 | 0 (0～0) |
| **Urinary dysfunction** | 3.40±3.65 | 2 (0～5) | Heat intolerance | 0.35±0.73 | 0 (0～0) |
| Urinary urgency | 0.62±0.91 | 0 (0～1) | **Pupillomotor dysfunction** | 0.22±0.57 | 0 (0～0) |
| Urinary incontinence | 0.24±0.59 | 0 (0～0) | Oversensitive to bright light | 0.22±0.57 | 0 (0～0) |
| Incomplete emptying | 0.42±0.77 | 0 (0～1) | **Sexual dysfunction** | 0.46±0.90 | 0 (0～0) |
| Weak stream of urine | 0.28±0.65 | 0 (0～0) | Men | 0.47±0.91 | 0 (0～0) |
| Frequency | 0.51±0.86 | 0 (0～1) | Women | 0.45±0.88 | 0 (0～0) |
| Nocturia | 1.34±1.21 | 1 (0～3) | **Total score** | 9.21 ± 7.39 | 8 (3～13) |

*Median (25% ~ 75% interquartile)

Abbreviations: AutD, Autonomic Dysfunction; SCOPA-AUT, Scale for Outcomes in Parkinson's disease for Autonomic symptoms.
