# Supplementary material for: Characteristics of Autonomic Dysfunction in Parkinson’s Disease: A Large Chinese Multicenter Cohort Study
Source: Front Aging Neurosci. 2021 Nov 30;13:761044. doi: 10.3389/fnagi.2021.761044 (PMC8670376; doi:10.3389/fnagi.2021.761044)
Supplement: Supplementary file 4 [file Table_3.DOCX]

**Supplementary Table 3. Comparison of NMSS subdomains with AutD**

| **Domains of NMSS** | **Without AutD (n=223)** | **Single-Domain AutD**  **(n=387)** | **Multiple-Domain AutD (n=1946)** | ***p*-value** |
| --- | --- | --- | --- | --- |
| Cardiovascular | 0.30±0.89 | 0.38±1.18 | 1.10±2.09 | **<0.001** |
| Sleep/fatigue | 4.45±4.95 | 5.57±5.92 | 10.16±7.82 | **<0.001** |
| Mood | 3.75±7.72 | 4.25±7.25 | 7.19±9.77 | **<0.001** |
| Perceptual problems | 0.29±1.06 | 0.38±1.18 | 1.18±2.76 | **<0.001** |
| Attention/memory | 1.85±3.53 | 2.09±3.37 | 3.51±4.11 | **<0.001** |
| Gastrointestinal | 1.17±3.13 | 2.45±4.03 | 5.20±5.09 | **<0.001** |
| Urinary | 1.51±3.52 | 2.74 ± 4.85 | 6.78 ± 6.68 | **<0.001** |
| Sexual function | 0.04±0.33 | 0.11±0.74 | 0.47±2.08 | **0.004** |
| Miscellaneous | 2.26±3.32 | 3.26±4.19 | 5.63±5.46 | **<0.001** |
| NMSS Total Score | 16.04±19.51 | 21.70±18.04 | 41.22±27.39 | **<0.001** |

Data were expressed as mean ± SD. Abbreviations: NMSS, Non-Motor Symptoms Scale; AutD, Autonomic Dysfunction.
