# Supplementary material for: Characteristics of Autonomic Dysfunction in Parkinson’s Disease: A Large Chinese Multicenter Cohort Study
Source: Front Aging Neurosci. 2021 Nov 30;13:761044. doi: 10.3389/fnagi.2021.761044 (PMC8670376; doi:10.3389/fnagi.2021.761044)
Supplement: Supplementary file 5 [file Table_4.DOCX]

**Supplementary Table 4. Comparison of each AutD symptom based on disease duration subgroups**

| **Items** | **DD≤3**  **（n=1120）** | **3<DD≤8**  **（n=941）** | **8<DD**  **（n=495）** | ***p*-value** |
| --- | --- | --- | --- | --- |
| **Gastrointestinal dysfunctions (total score)** | 2.45±2.79 | 3.45±3.23 | 4.70±3.53 | **<0.001** |
| Difficulty swallowing/choked | 0.19±0.47 | 0.32±0.60 | 0.46±0.68 | **<0.001** |
| Sialorrhea | 0.50±0.78 | 0.70±0.85 | 0.96±0.97 | **<0.001** |
| Dysphagia | 0.09±0.34 | 0.15±0.45 | 0.22±0.53 | **<0.001** |
| Early abdominal fullness | 0.14±0.47 | 0.19±0.53 | 0.16±0.48 | 0.078 |
| Constipation | 0.72±1.07 | 1.01±1.19 | 1.42±1.26 | **<0.001** |
| Straining for defecation | 0.78±1.07 | 1.07±1.17 | 1.43±1.23 | **<0.001** |
| Fecal incontinence | 0.03±0.23 | 0.03±0.21 | 0.04±0.23 | 0.201 |
| **Urinary dysfunctions (total score)** | 2.68±3.32 | 3.72±3.67 | 4.43±4.01 | **<0.001** |
| Urinary urgency | 0.48±0.83 | 0.69±0.95 | 0.79±0.96 | **<0.001** |
| Urinary incontinence | 0.17±0.50 | 0.26±0.60 | 0.38±0.73 | **<0.001** |
| Incomplete emptying | 0.33±0.70 | 0.43±0.77 | 0.58±0.90 | **<0.001** |
| Weak stream of urine | 0.21±0.59 | 0.27±0.63 | 0.43±0.78 | **<0.001** |
| Frequency | 0.37±0.75 | 0.61±0.91 | 0.65±0.93 | **<0.001** |
| Nocturia | 1.13±1.18 | 1.45±1.22 | 1.60±1.17 | **<0.001** |
| **Cardiovascular dysfunctions (total score)** | 0.47±1.08 | 0.57±1.20 | 0.57±1.15 | 0.126 |
| Light-headed when standing up | 0.25±0.58 | 0.31±0.64 | 0.33±0.65 | **0.024** |
| Light-headed when standing for some time | 0.20±0.52 | 0.23±0.57 | 0.22±0.56 | 0.596 |
| Syncope | 0.03±0.19 | 0.04±0.22 | 0.02±0.16 | 0.278 |
| **Thermoregulatory dysfunctions (total score)** | 1.10±1.86 | 1.46±2.06 | 1.74±2.19 | **<0.001** |
| Hyperhidrosis during the day | 0.33±0.72 | 0.46±0.83 | 0.57±0.90 | **<0.001** |
| Hyperhidrosis during the night | 0.19±0.56 | 0.28±0.68 | 0.34±0.72 | **<0.001** |
| Cold intolerance | 0.31±0.70 | 0.34±0.71 | 0.35±0.73 | 0.143 |
| Heat intolerance | 0.28±0.66 | 0.38±0.74 | 0.48±0.84 | **<0.001** |
| **Pupillomotor dysfunctions (total score)** | 0.21±0.54 | 0.24±0.60 | 0.23±0.56 | 0.453 |
| Oversensitive to bright light |  |  |  |  |
| **Sexual dysfunctions (total score)** | 0.40±0.85 | 0.51±0.93 | 0.50±0.92 | **0.008** |
| Men | 0.39±0.85 | 0.50±0.93 | 0.58±0.99 | **0.008** |
| Women | 0.41±0.86 | 0.52±0.94 | 0.40±0.82 | 0.100 |
| **Total score** | 7.30±6.56 | 9.94±7.52 | 12.16±7.73 | **<0.001** |

Data were expressed as mean ± SD. Abbreviations: AutD, Autonomic Dysfunction; DD, Disease Duration.
