# Supplementary material for: Characteristics of Autonomic Dysfunction in Parkinson’s Disease: A Large Chinese Multicenter Cohort Study
Source: Front Aging Neurosci. 2021 Nov 30;13:761044. doi: 10.3389/fnagi.2021.761044 (PMC8670376; doi:10.3389/fnagi.2021.761044)
Supplement: Supplementary file 6 [file Table_5.DOCX]

**Supplementary Table 5. Comparison of each AutD symptom based on age of onset subgroups**

| **Items** | **AOO≤50（n=804）** | **50<AOO≤60**  **（n=875）** | **60<AOO≤70**  **（n=681）** | **70<AOO**  **（n=196）** | ***p-value*** |
| --- | --- | --- | --- | --- | --- |
| **Gastrointestinal dysfunctions** | 2.62±3.14 | 3.24±3.14 | 3.76±3.24 | 4.15±3.32 | **＜0.000** |
| Difficulty swallowing/choked | 0.26±0.55 | 0.28±0.57 | 0.33±0.59 | 0.33±0.63 | **0.048** |
| Sialorrhea | 0.57±0.85 | 0.66±0.83 | 0.75±0.90 | 0.79±0.89 | **＜0.000** |
| Dysphagia | 0.12±0.43 | 0.13±0.42 | 0.15±0.43 | 0.14±0.45 | 0.252 |
| Early abdominal fullness | 0.15±0.47 | 0.16±0.51 | 0.16±0.48 | 0.21±0.57 | 0.620 |
| Constipation | 0.74±1.11 | 0.96±1.17 | 1.13±1.22 | 1.29±1.20 | **＜0.000** |
| Straining for defecation | 0.76±1.09 | 1.02±1.16 | 1.20±1.20 | 1.35±1.19 | **＜0.000** |
| Fecal incontinence | 0.03±0.20 | 0.03±0.21 | 0.04±0.26 | 0.05±0.26 | 0.169 |
| **Urinary dysfunctions** | 2.76±3.37 | 3.45±3.69 | 4.01±3.92 | 3.66±3.28 | **＜0.000** |
| Urinary urgency | 0.51±0.86 | 0.61±0.91 | 0.75±0.98 | 0.66±0.87 | **＜0.000** |
| Urinary incontinence | 0.18±0.51 | 0.25±0.62 | 0.29±0.64 | 0.30±0.62 | **0.001** |
| Incomplete emptying | 0.36±0.72 | 0.42±0.78 | 0.47±0.80 | 0.45±0.77 | **0.025** |
| Weak stream of urine | 0.22±0.56 | 0.30±0.69 | 0.34±0.72 | 0.20±0.56 | **0.003** |
| Frequency | 0.41±0.78 | 0.50±0.86 | 0.64±0.93 | 0.49±0.84 | **＜0.000** |
| Nocturia | 1.08±1.16 | 1.38±1.21 | 1.52±1.22 | 1.56±1.19 | **＜0.000** |
| **Cardiovascular dysfunctions** | 0.38±0.93 | 0.52±1.17 | 0.66±1.27 | 0.70±1.25 | **＜0.000** |
| Light-headed when standing up | 0.22±0.53 | 0.27±0.61 | 0.36±0.71 | 0.35±0.64 | **＜0.000** |
| Light-headed when standing  for some time | 0.15±0.44 | 0.21±0.55 | 0.26±0.62 | 0.32±0.65 | **＜0.000** |
| Syncope | 0.02±0.14 | 0.04±0.23 | 0.03±0.20 | 0.03±0.20 | 0.234 |
| **Thermoregulatory dysfunctions** | 1.36±2.04 | 1.40±2.04 | 1.34±2.02 | 1.14±1.82 | 0.410 |
| Hyperhidrosis during the day | 0.42±0.80 | 0.46±0.82 | 0.41±0.79 | 0.32±0.72 | 0.130 |
| Hyperhidrosis during the night | 0.27±0.67 | 0.23±0.61 | 0.22±0.62 | 0.33±0.71 | **0.044** |
| Cold intolerance | 0.33±0.70 | 0.35±0.74 | 0.33±0.73 | 0.23±0.60 | 0.155 |
| Heat intolerance | 0.34±0.72 | 0.36±0.74 | 0.38±0.75 | 0.26±0.61 | 0.241 |
| **Pupillomotor dysfunctions** | 0.20±0.53 | 0.24±0.60 | 0.21±0.56 | 0.27±0.62 | 0.656 |
| Oversensitive to bright light | 0.20±0.53 | 0.24±0.60 | 0.21±0.56 | 0.27±0.62 | 0.656 |
| **Sexual dysfunctions** | 0.51±0.92 | 0.43±0.88 | 0.45±0.90 | 0.45±0.85 | 0.254 |
| Men | 0.61±1.00 | 0.38±0.84 | 0.41±0.88 | 0.44±0.83 | **<0.000** |
| Women | 0.37±0.80 | 0.48±0.91 | 0.48±0.92 | 0.47±0.87 | 0.362 |
| **Total score** | 7.83±7.26 | 9.28±7.33 | 10.42±7.57 | 10.37±6.64 | **＜0.000** |

Data were expressed as mean±SD. Abbreviations: AutD, Autonomic Dysfunction; AOO, Age of onset.
