# Supplementary material for: Characteristics of Autonomic Dysfunction in Parkinson’s Disease: A Large Chinese Multicenter Cohort Study
Source: Front Aging Neurosci. 2021 Nov 30;13:761044. doi: 10.3389/fnagi.2021.761044 (PMC8670376; doi:10.3389/fnagi.2021.761044)
Supplement: Supplementary file 7 [file Table_6.DOCX]

**Supplementary Table 6. Multicollinearity Diagnosis of Logistic Regression Model**

| **Variables** | **VIF** |
| --- | --- |
| Age | 8.301 |
| Gender | 1.618 |
| BMI | 7.167 |
| Age at onset | 2.653 |
| Duration of disease | 1.537 |
| LEDD | 1.196 |
| UPDRS total score | 2.172 |
| Motor subtypes | 1.083 |
| H&Y stage | 1.589 |
| Dyskinesia | 1.185 |
| FOG | 1.223 |
| Cognition impairment | 1.104 |
| RBD | 1.188 |
| EDS | 1.162 |
| PDSS score | 1.385 |
| Hyposmia | 1.057 |
| Depression | 1.476 |
| RLS | 1.016 |
| Fatigue | 1.262 |
| PDQ-39 score | 2.340 |

Abbreviations: VIF, Variance Inflation Factor; BMI, Body Mass Index; LEDD, Levodopa Equivalent Daily Dose; UPDRS, Unified Parkinson’s disease Rating Scale; H&Y, Hoehn and Yahr scale; FOG, freezing of gait; RBD, Rapid Eye Movement Sleep Behavior Disorder; EDS, Excessive Daytime Sleepiness; PDSS, Parkinson’s disease Sleep Scale; RLS, Restless legs syndrome; PDQ-39, Parkinson’s disease questionnaire-39 item version.
