# Supplementary material for: Characteristics of Autonomic Dysfunction in Parkinson’s Disease: A Large Chinese Multicenter Cohort Study
Source: Front Aging Neurosci. 2021 Nov 30;13:761044. doi: 10.3389/fnagi.2021.761044 (PMC8670376; doi:10.3389/fnagi.2021.761044)
Supplement: Supplementary file 8 [file Table_7.DOCX]

Supplementary Table 7: Variables not in the logistic regression model.

| **Variables** | **Odds ratio** | **95% CI** | ***p-value*** |
| --- | --- | --- | --- |
| Gender ratio (male vs female) | 1.288 | 0.826 - 2.009 | 0.265 |
| BMI | 0.979 | 0.875 - 1.097 | 0.720 |
| Age at onset (LOPD vs EOPD) | 1.077 | 0.604 - 1.92 | 0.802 |
| Disease duration | 1.031 | 0.974 - 1.092 | 0.290 |
| LEDD | 1.000 | 1.000 - 1.001 | 0.416 |
| Motor subtypes |  |  | 0.763 |
| (Intermediate vs TD) | 1.024 | 0.611 - 1.716 | 0.928 |
| (PIGD vs TD) | 1.152 | 0.782 - 1.698 | 0.475 |
| H&Y stage (>3 vs ≤2.5) | 0.871 | 0.486 - 1.561 | 0.642 |
| Dyskinesia (with vs without) | 1.242 | 0.691 - 2.232 | 0.470 |
| FOG (with vs without) | 1.393 | 0.806 - 2.408 | 0.236 |
| Cognition impairment (with vs without) | 1.319 | 0.615 - 2.828 | 0.477 |
| EDS (with vs without) | 1.383 | 0.84 - 2.277 | 0.203 |
| PDSS | 0.999 | 0.991 - 1.007 | 0.797 |
| RLS (with vs without) | 1.114 | 0.589 - 2.11 | 0.740 |
| PDQ-39 | 1.002 | 0.989 - 1.016 | 0.716 |

Abbreviations: LOPD, Late onset Parkinson’s disease; EOPD, Early onset Parkinson’s disease; BMI, Body Mass Index; LEDD, Levodopa Equivalent Daily Dose; UPDRS, Unified Parkinson’s disease Rating Scale; TD, Tremor-Dominant; PIGD, Postural Instability and Gait Difficulty; H&Y, Hoehn and Yahr; FOG, freezing of gait; EDS, Excessive daytime sleepiness; PDSS, Parkinson’s disease Sleep Scale; RLS, Restless legs syndrome; PDQ-39, Parkinson’s disease questionnaire-39 item version.
